# Maternal depression is associated with child undernutrition: A cross‐sectional study in Ethiopia

**DOI:** 10.1111/mcn.12934

**Published:** 2019-12-12

**Authors:** Anchamo Anato, Kaleab Baye, Zelalem Tafese, Barbara J. Stoecker

**Affiliations:** ^1^ School of Nutrition, Food Science and Technology Hawassa University Ethiopia; ^2^ Center for Food Science and Nutrition Addis Ababa University Addis Ababa Ethiopia; ^3^ Department of Nutritional Sciences Oklahoma State University Stillwater Oklahoma USA

**Keywords:** child undernutrition, inappropriate complementary feeding, maternal depression, northern Ethiopia, stunting

## Abstract

Child undernutrition is widespread in low‐ and middle‐income countries (LMIC) and is associated with health and economic losses. Undernutrition is estimated to contribute to 3.1 million deaths per year in children less than 5 years of age. A complex causal and contextual factors contributing to child undernutrition have been assessed, but maternal depression, which could contribute to child undernutrition by interfering with the mother's child caring practice and ability, has been received little attention. The objective of this study was to assess the association between maternal postpartum depression symptoms and infant (5–10 months of age) stunting in northern Ethiopia. A community‐based cross‐sectional study was conducted among mother–infant pairs (*n* = 232) between March and April 2018. Through interviewer‐administrated questionnaire, information on sociodemographic variables were collected, and maternal depression symptoms were assessed using the Edinburgh Postnatal Depression Scale (EPDS≥13). Infants' length and weight were measured and converted to length and weight for age Z scores using the WHO growth standards. Breastfeeding was a norm, but the adequacy of complementary feeding practice was sub‐optimal. Only 25% of the infants met the minimum meal frequency (MMF), less than 10% met the minimum dietary diversity (MMD; 9%) or minimum acceptable diet (7%). Maternal depression was prevalent (22.8%) and was significantly associated with inappropriate complementary feeding and stunting (*P* < .05). Improving complementary feeding practices is central to preventing stunting in this and other settings. However, such efforts should integrate interventions that address maternal depression to improve child feeding and caring practices to effectively prevent stunting.

**Key messages**

- Maternal depression especially postnatal is a strong predictor of infants' undernutrition measured as stunting and underweight.
- Maternal depression is strongly associated with inappropriate complementary feeding practices of infants.
- Increased postnatal care visits should be promoted, and maternal depression awareness should be integrated in maternal and infant health programmes like health extension packages and other preventive programmes aiming to improve the care and feeding practices of the mother for her infant.
- Maternal mental health care and treatment guidelines especially for postnatal depression are crucial, and attention to maternal mental health is recommended for all countries because of its public health significance.

Future longitudinal study is recommended to evaluate the potential cause–effect relationship between addressing maternal depression and infants' undernutrition in rural areas of low income countries.

## 1 INTRODUCTION

Child undernutrition is a global public health problem causing both health and economic losses for nations and contributing to 3.1 million deaths annually in children under 5 years of age (Black et al., 2013). The first 1000 days of life, from conception to the child's second birthday, is a critical period characterized by rapid physical and mental development that requires high energy and nutrient intake. Poor breastfeeding and complementary feeding practices in LMIC have been associated with undernutrition, with largely irreversible consequences leading to compromised cognitive development, poor productivity and lower earnings in later life (Black et al., 2008).

One of the highest prevalence of undernutrition is in Sub‐Saharan Africa, where 39.4% of children under 5 are stunted, 24.9 % are underweight and 10.3 % are wasted (UNICEF et al. 2018). Ethiopia has made remarkable progress in stunting reduction over the past decades, but levels of stunting (38%) remain one of the highest in Sub‐Saharan Africa (CSA, 2016). Several studies, using the Demographic and Health Surveys and other nationally representative data, have investigated the determinants of stunting in Ethiopia. These efforts have shown that a large proportion of stunting remains unexplained, suggesting that these routine data are missing critical causal factors that may contribute to the persistence of high levels of stunting (Headey, 2014; Woodruff et al., 2017). One such factor could be the state of maternal mental health, particularly postpartum depression.

Post‐partum depression is a common psychotic disorder linked to physical, emotional and behavioural changes affecting women in the first post‐partum year. Maternal depression symptoms have been reported to contribute to child undernutrition (Black, Baqui, Zaman, Arifeen, & Black, 2009) as it can interfere with the mother's sense of responsibility and child caring practices (Kakyo, Muliira, Mbalinda, Kizza, & Muliira, 2012; Surkan, Kennedy, Hurley, & Black, 2011), leading to a mother's inability to provide adequate diet for the child. Stewart et al. (2008) reported that infants in sub‐Saharan Africa whose mothers' were affected by common mental disorder were more likely to be stunted but not underweight. However, the few studies that investigated association between maternal depression symptoms and child undernutrition in Ethiopia produced mixed results. For example, Nguyen et al. (2013) reported that maternal common mental disorders were associated with undernutrition in Bangladesh and Vietnam but not in Ethiopia. However, this and other studies evaluated mental disorders but not specifically maternal depression symptoms. They also included a wider age group (6–59 months), which could underestimate the effect of maternal depression symptoms as the highest need for maternal care is in the first 2 years coinciding with the time that undernutrition reaches a peak. Particularly, postnatal depression could be highest in the child's first year of life. Therefore, the aim of this study was to assess the association between maternal depression, specifically postnatal depression, and undernutrition in infants aged 5–10 months old in northern Ethiopia.

## 2 METHODS

### 2.1 Study site

This community‐based cross‐sectional study was conducted between March and April 2018 in rural areas of northern Ethiopia. The region is characterized by high prevalence of child undernutrition, with a stunting prevalence of 46%, which is above the national average (38%) (CSA). The study was conducted in 10 kebeles (smallest administration unit) in the Meket district.

### 2.2 Sample size and sampling procedures

A total of 238 households targeted for the Ethiopian government's Productive Safety Net Program (PSNP_4_) were included in this study using a multi‐stage sampling technique. Of the 27 kebeles in Meket district, 10 were randomly selected. A listing of PSNP_4_ households with infants 5–10 months of age was compiled through house‐to‐house visits, and a random sample in proportion to the size of the kebeles was selected. Being resident in the district and having an infant 5–10 months old were the eligibility criteria for participating in the study. The interviewees were mothers with infants 5–10 months.

### 2.3 Data collection

#### 2.3.1 Sociodemographic characteristics

Data were collected by face‐to‐face interviews with the mother in a quiet room at the health post. Basic sociodemographic data such as age of the mother, marital status, estimated household's average monthly income, religion, educational status and occupation as well as other data concerning household economic status were collected.

#### 2.3.2 Household food insecurity access scale

The Household Food Insecurity Access Scale (HFIAS) (Coates, Swindale, & Bilinsky, 2007) was used to assess household food insecurity during the 4 weeks preceding the survey. The households were categorized into four groups: food secure, mildly food insecure, moderately food insecure and severely food insecure. Finally, these were merged in to two groups: food secure and food insecure (mildly, moderately and severely) households.

#### 2.3.3 Anthropometric measures

All anthropometric measurements were taken by the lead author. Length was measured to the nearest 0.1 cm using a portable wooden recumbent length board with a fixed head and sliding foot piece. Weight was measured to the nearest 0.01 kg, with an electronic scale (UNICEF Seca 770) with light clothing. Measurements were done in duplicate, and the mean value was calculated. Infant's age was recorded from the immunization card or birth certificate. However, in the absence of such documents, maternal recall with a calendar of events was used. Infant anthropometric status was calculated as length for age z‐score (LAZ), weight for length z‐score (WLZ) and weight for age z‐score (WAZ) (WHO, 2006) reference data.

#### 2.3.4 Household socioeconomic status

Household wealth index (low, medium and high) was constructed using principal component analysis (PCA). Selected household assets, ownership and number of livestock, materials used to build house and agricultural land size were used to calculate the index. The factor scores used explained 68.4% of the total variance in the household's wealth index.

#### 2.3.5 Maternal workload

PCA was also used to calculate maternal workload. The 10 distinct laborious activities in the community in the preceding 1 week of the survey were included in the analysis. The variables included in the analysis were collecting firewood and fetching water (walking distance > 30 min), washing clothes, land preparation, weeding, manual mowing, threshing, grinding and pounding grain and terracing. The final components which explained 70.6% of the total variance were retained and women were classified into three categories (low, medium and heavy) of workload based on their scores.

#### 2.3.6 Infant feeding practices

Data on infant feeding practices were assessed using the WHO IYCF indicators (WHO, 2008). Early initiation of breastfeeding, exclusive and continued breastfeeding, introduction to complementary foods and complementary feeding indicators such as MMF, MMD and minimum adequate diet were assessed. Foods consumed in the 24 h prior the survey were categorized into the following seven food groups to calculate the dietary diversity score: (i) grains, roots and tubers; (ii) legumes and nuts; (iii) dairy products; (iv) eggs; (v) flesh foods (meat, fish, poultry and organ meats); (vi) vitamin A‐rich fruits and vegetables; and (vii) other fruits and vegetables. The proportion of breastfed infants meeting the MMF (≥3) and the MMD score (≥4) was calculated.

#### 2.3.7 Health‐seeking behaviour and practice

The place of delivery of the infant and immunization status was recalled by the mother, and whenever available was confirmed by checking the immunization cards. Infant illness (morbidity) and healthcare‐seeking practices of the mother were assessed through maternal recall of signs and symptoms related to acute respiratory infections, diarrhoea, cough and/or cold and fever over the 2 weeks prior the survey.

#### 2.3.8 Maternal depression assessment

Maternal depression was assessed using the Edinburgh Postnatal Depression Scale (EPDS) (Cox, Holden, & Sagovsky, 1987) which included 10 items. Mothers rated the extent to which each item matched their feelings during the 7 days prior to the survey. Possible scores ranges from 0 to 30, with higher scores indicating greater severity of depression. A score of 13 and above indicated the postnatal depression. The EPDS has been used as a screening tool for detecting postnatal depression in different cultures (Kakyo et al., 2012) and has been used in several African countries (Madeghe, Kimani, Stoep, Nicodimos, & Kumar, 2016; Ukaegbe, Iteke, Bakare, & Agbata, 2012; Yator, Mathai, der Stoep, Rao, & Kumar, 2016). A validation study in Ethiopia also demonstrated the acceptability and utility of the Amharic version for use as a screening tool for postnatal depression (Tesfaye, Hanlon, Wondimagegn, & Alem, 2009). The internal consistency measured by Cronbach's alpha (0.80) was acceptable.

### 2.4 Data processing and analysis

Data were checked, coded and entered into SPSS, v. 20, for analysis. Categorical data were analysed by descriptive statistics (frequency and percentage), whereas range, mean and standard deviations were used to present continuous variables. The normal distribution of the data was checked with the Kolmogorov‐Smirnov test (Mishra et al., 2019).

Bivariate analyses were conducted, and all variables with *P* < .25 were included in multivariate logistic regression. The model fit was checked using the Hosmer–Lemeshow goodness of fit test. Statistical significance was considered at the 95% confidence interval, and both crude and adjusted odds ratios are reported.

## 3 RESULTS

### 3.1 Sociodemographic and anthropometric characteristic

Among the 238 recruited mother–infant pairs, 232 completed the study (97.5% response rate). Almost half (49.1%) of the study mothers were in the 25–34 year range, and 95.3% were married (Table 1). Over half (59 %) of the mothers had no formal education, and 83.2% were unemployed. Nearly half of the participants (48.7 %) were from food insecure households. About a third (30.6%; 95% CI: 24.6–36.6) of infants were stunted; 20.7% (95%CI: 15.5–25.9) were underweight, and 7.8% (95% CI: 4.3‐11.2) were wasted.

**Table 1:** Sociodemographic characteristics and anthropometric measures of participants (*n* = 232) in northern Ethiopia, March–April, 2018

Variables (*n* = 504)	Percentage (%) or mean (SD)
Age of mother	28.0 (6.6)
Level of education	
Not educated	59.1
Primary and above	40.9
Occupation	
Housewife	83.2
Employed*	16.8
Marital status	
Married	95.7
Widow/separated	4.3
Monthly household income (ETB)	
≤150	59.5
151–650	20.7
≥651	19.8
Household size	4.97 (1.56)
Wealth index	
Low	32.8
Medium	50.4
High	16.8
Child age (month)	6.31 (1.07)
Owns sanitary facility (latrine)	84.1
Owns milk cows	40.1
Owns chickens	45.3
Level of household food security	
Food secure	51.3
Food insecure	48.7
Infant anthropometric status	
Stunted	30.6
Underweight	20.7
Wasted	7.8
LAZ	‐1.09±1.46
WAZ	‐0.92±1.25
WLZ	‐0.27±1.29

Abbreviations: LAZ, length for age z‐score; SD, standard deviation; WAZ, weight for age z score; WLZ, weight for length z score.

* Retailers/government employee/causal work/own business.

### 3.2 Breast feeding and complementary feeding practices

Early breast feeding practices were recalled by mothers. Amongst the studied mothers, breast feeding initiation within an hour of birth was 77.2% and 95.7% of mothers currently breast feeding (Table 2). Complementary food intakes of infants were assessed and analysed for infants aged 6–10 months (*n* = 198). Although a number of infants were not yet being fed complementary foods, cereals/grains was the most commonly consumed food group (35.9%) and only a very few infants (5.1%) had consumed meat in the 24 h before the survey. Most infants (81.5%) had low dietary diversity (0–2 food groups from 7 groups) in addition to breast milk. Three‐fourths (74.7%) of infants aged 6–10 months did not meet recommendations for minimum number of meals during the day before the survey.

**Table 2:** Breastfeeding (*n* = 232) and complementary feeding practices (*n* = 198) of infants aged 5–10 months in northern Ethiopia, March–April 30, 2018

Feeding practices	Percentage (%)
Fed colostrum^a^	86.6
Early initiation of breast feeding (within 1 h of birth)^b^	77.2
Currently breast feeding	95.7
Timely introduction of complementary feeding	27.2
Cereal/grains	35.9
Other fruits and vegetables	5.6
Vitamin A‐rich fruits and vegetables	9.7
Egg	11.1
Meat, organ meat, fish and poultry	5.1
Legumes, nuts and seeds	25.4
Milk and milk product	19.7
Mean number of food groups consumed	1.12±1.56
DDS^c^	
High (5–7)	4.7
Medium (3–4)	13.8
Low (0–2)	81.5
Met minimum meal frequency (6–10 months)^d^	25.3
Met minimum dietary diversity (6–10 months)^e^	9.1
Met minimum acceptable diet (6–10 months)^f^	6.9

a First milk (thick yellowish milk the mother produces for the first few days after birth).

b Proportion of children who were put to breast within 1 h of birth.

c Dietary diversity score.

d Proportion of breast‐fed children (6–10 months) who received solid, semisolid or soft foods, a minimum of 2 times for children 6 to 8 months and 3 times for children 9 to 10 months.

e Proportion of children aged 6 to 10 months who received from 4 or more food groups (of the 7) during the previous day.

f Proportion of children aged 6 to 10 months who had both the minimum meal frequency and minimum dietary diversity on the previous day.

### 3.3 Maternal workload, depression symptoms and health‐seeking behaviours

A third of mothers had a heavy workload, 22.8% (95% CI: 17.7–28.4) had symptoms of depression based on EPDS score ≥13 (Table 3). More than half (53.9%) of mother reported home delivery, and 82.3% had antenatal care at least once during their most recent pregnancy. Only 81% of infants had received any vaccinations. Nearly a quarter (24.1%) of infants had symptoms of acute respiratory infection, and almost 30% had diarrhoea during the previous 2 weeks. A large proportion (53.9%) of infants had symptoms of cough/cold.

**Table 3:** Maternal workload, maternal depression, health‐seeking behaviours and infant illness in northern Ethiopia, March–April, 2018

Variables	Percentage (%)
Maternal workload	
Low	34.1
Medium	32.8
Heavy	33.2
Maternal depression symptoms	
EPDS≥13	22.8
Place of delivery	
Health facility	46.1
Home	53.9
Received ANC at least once	82.3
Received PNC at least once	53.3
Received counselling from health professional during ANC visits	72.0
Infant illness	
Diarrhoea	29.7
Acute respiratory infection	24.1
Fever	40.1
Cough/cold	53.9
Infant received any vaccinations for his/her age	81.0

### 3.4 Factors associated with infants' stunting and complementary feeding

Lack of maternal education (*P* = .02) and maternal depression symptoms (*P* = .009) predicted stunting, whereas being in the upper wealth category was inversely associated (*P* = .049) with stunting in bivariate analyses (Table 4). In fact, maternal depression symptoms (COR = 3.27, 95% CI: 1.06–10.07), household food insecurity (COR = 3.32, 95% CI: 1.01–10.99) and lack of maternal education (COR = 5.28, 95% CI: 1.30–21.37) were associated with inappropriate complementary feeding in bivariate analyses (Table 5). However, in multivariate logistic regression that adjusted for covariates, only maternal depression symptoms and lack of maternal education remained statistically significant. The AOR inappropriate complementary feeding among mothers with depression was 3.67 (95% CI: 1.09–12.32), whereas the AOR for mothers with no formal education was 5.14 (95 % CI: 1.24‐21.21).

**Table 4:** Results of bivariate and multivariate logistic regression models predicting the likelihood of infant stunting

Variables	COR (95% CI)	*P* value[Link]	AOR (95% CI)	*P* value[Link]
Maternal depression				
EPDS score <13^a^				
EPDS score ≥13	2.32 (1.23–4.38)	.009	2.55 (1.24–5.25)	.011
Wealth index				
Lower^a^				
Middle	0.81 (0.36–1.82)	.61		
Upper	0.53 (0.28–0.99)	.049		
Level of education				
Primary and above^a^				
Not educated	2.02 (1.11–3.67)	.02	2.05 (1.08–3.93)	.031
Occupation				
Housewife^a^				
Working outside	0.53 (0.23–1.22)	.13		
Level of household food security				
Food secure^a^				
Food insecure	1.47 (0.56–3.87)	.42		
Maternal workload				
Low^a^				
Medium	1.2 (0.54–2.12)	.83		
Heavy	1.01 (0.51–2.01)	.91		

Abbreviations: AOR, adjusted odd ratio; COR, crude odd ratio.

^*^Statistically significant *P* < .05.

a Reference categories.

**Table 5:** Results of bivariate and multivariate logistic regression analyses of association between maternal depression and inappropriate complementary feeding practices of infants^b^

Variables	COR (95% CI)	*P* value[Link]	AOR (95% CI)	*P* value[Link]
Maternal depression				
EPDS score <13^a^				
EPDS score ≥13	3.27 (1.06–10.07)	.039	3.67 (1.09–12.32)	.035
Wealth index				
Upper^a^				
Middle	2.53 (0.30–21.42)	.39		
Lower	3.73 (0.34–32.43)	.23		
Level of education				
Educated^a^				
Not educated	5.28 (1.30–21.37)	.019	5.14 (1.24–21.21)	.023
Occupation				
Housewife^a^				
Working	0.53 (0.04–3.32)	.37		
Family size				
<4^a^				
4–5	0.87 (0.21–3.66)	.85		
>5	1.05 (0.31–3.64)	.92		
Level of household food security				
Food secure^a^				
Food insecure	3.32 (1.01–10.99)	.049		
Maternal workload				
Low^a^				
Medium	1.8 (0.41–7.88)	.43		
Heavy	2.5 (0.59–10.47)	.25		

Abbreviations: AOR, adjusted odd ratio; COR, crude odd ratio.

^*^Statistically significant *P* < .05.

a Reference categories.

b If infant did not meet WHO composed indicators (timely introduction of semi‐solid or solid complementary feeding, minimum dietary diversity and minimum meal frequency).

## 4 DISCUSSION

Our findings highlight the relatively good practice of continued breastfeeding but also illustrate the very poor complementary feeding practices characterized by low dietary diversity and meal frequency. Poor complementary feeding practices, low socioeconomic status and lack of maternal education are common predictors of infant stunting, but maternal depression symptoms also was found to be associated with inappropriate complementary feeding and stunting.

The first 1000 days from conception to the child's second birthday is considered to be a critical window of opportunity for nutrition that, if missed, leads to grave consequences such as impaired cognitive function, overall poor health and loss of future productivity (Cursik & Georgieff, 2016; Hoddinott, Alderman, Behrman, Haddad, & Horton, 2013). Considering the importance of the first 1000 days, WHO/FAO developed child feeding guidelines and indicators to routinely monitor and support programmes that aim to improve nutrition in this critical period (WHO, 2010). National nutrition policies are also focusing on this critical window of opportunity, and, consequently, a number of prioritized interventions (e.g. nutrition education, fortification and vitamin A supplementation) are being implemented (Bhutta et al., 2013; WHO, 2016).

In the present study that only focused in the first 1 year of life, one‐third of the children were already stunted. Although continued breastfeeding was a norm, inappropriate complementary feeding practices were widespread. Consumption of nutrient‐dense food groups such as animal source foods and vitamin A‐rich fruits and vegetables was very low. This is unfortunate, as dietary diversity in general and animal source food consumption in particular are consistently associated with lower risk of stunting (Kraseve, An, Kumapley, Bégin, & Frongillo, 2017). Poor knowledge about appropriate complementary feeding practices, low production diversity and availability, poor market access and unaffordability of nutrient‐dense foods are among possible constraints limiting optimal complementary feeding practices (Abay & Hirvonen, 2017; Hirvonen, Hoddinott, Minten, & Stifel, 2017; Muehlhoff et al., 2017; Ruel, 2019).

Indeed, in this and other similar LMIC settings, socioeconomic status and maternal education were found associated with complementary feeding practices (Fein, Labiner‐Wolfe, Scanlon, & Grummer‐Strawn, 2008). Nutrition education through the health extension programme in Ethiopia has been shown to improve knowledge and practice of complementary feeding to varying extents (Gebremedhin et al., 2017). This high variability in impact can be related to inconsistent levels of counseling skills of HEWs but could also be determined by the aptitude of caregivers to integrate and practice recommended actions. Although some studies have evaluated the effectiveness of knowledge‐sharing practices of HEWs (Abebe, Haki, & Baye, 2016), little emphasis has been given to maternal factors like maternal depression that can affect the extent to which recommended actions are practiced in households.

Maternal depression symptoms affected a fifth of the study participants and was significantly associated with both poor complementary feeding practices and stunting, even after controlling for covariates like socioeconomic status and maternal education. Post‐partum maternal depression is quite common in LMIC and prevalence of up to 46% has been reported in Bangladesh (Hagos, Shikur, Hanlon, & Lindtjørn, 2018; Khan & Flora, 2017). Such levels of maternal depression were previously found to compromise care and infant feeding practices, including breastfeeding and complementary feeding (Stewart et al., 2008), which can lead to stunting. Indeed, the association between maternal depression symptoms and child stunting highlighted by our study is supported by a recent multi‐country study that evaluated psychological risk factors among women and child stunting in 137 LMIC (Fawzi et al., 2019). This study used effects sizes from a meta‐analysis (Surkan et al., 2011) to estimate impact of maternal depression on stunting. However, given the observational nature of the evidence, more rigorous community‐based trials evaluating the impact of addressing maternal depression are warranted.

The present study has a number of limitations that need to be considered when interpreting our findings. First, the cross‐sectional nature of the study does not allow causal inferences to be made; hence, our findings relating maternal depression, complementary feeding practices and stunting should be considered as associations. However, these associations have strong theoretical justifications that could be confirmed through future experimental studies. Mental health issues other than depression are not addressed, and the causal factors behind the high proportion of maternal depression are not thoroughly addressed in this study. Future studies can help understand the causal factors leading to maternal depression and help design effective interventions to address it. However, a key strength of the present study is the uniquely comprehensive design that captured issues of infant feeding practices, maternal depression, mothers' workload, health‐seeking behaviours and child stunting.

Despite the above limitations, our study highlights that poor complementary feeding practices are common and that the stunting prevalence is high. Stunting was associated with commonly identified factors like maternal education and socioeconomic status, which were also predictors of optimal complementary feeding practice. However, a less commonly studied factor, maternal depression, was also found to be consistently associated with both inappropriate complementary feeding practices and stunting. This is an important finding that can have implications in the design and implementation of interventions that aim to improve infant and young child feeding and prevent stunting. Although community‐based trials are needed to establish the causal relationship between stunting and maternal depression, training HEWs in screening depression symptoms, routine monitoring and treatment of postnatal depression and integrating maternal mental health and stunting reduction interventions in a wider framework that promote early child development is critical.

## CONFLICTS OF INTEREST

The authors declare that they have no conflicts of interest.

## CONTRIBUTIONS

AA developed study design and supervised the field data collections. AA and BJS performed statistical analysis and interpretation of the data. AA wrote the first draft. KB, BJS, and ZT edited the draft manuscript. All authors have reviewed the final manuscript and approved the submission.
